# Assessment of the Distal Extent of the A1 Pulley Release: A New Technique

**Published:** 2008-08-22

**Authors:** Ron Hazani, Nitin J. Engineer, Linda L. Zeineh, Bradon J. Wilhelmi

**Affiliations:** Division of Plastic Surgery, School of Medicine, University of Louisville, Louisville, KY; None of the authors have any commercial association or financial disclosure that pose or create a conflict of interest with information presented in this article.

## Abstract

**Objective:** Sharp division of the A1 pulley is a time-honored technique for the treatment of flexor tendon entrapment; however, this procedure is not without complications. The anatomy of the A1 pulley system has been carefully investigated. Knowledge of superficial anatomic landmarks can assist with demarcating the distal edge of the A1 pulley and prevent damage to the critical A2 pulley. **Methods:** Nine fresh cadaveric hands were dissected with the aid of loupe magnification. On the basis of known anatomic landmarks of the proximal portion of the cruciate (C0) pulley, percutaneous placement of a 25-gauge needle 5 mm proximal to the palmar digital crease marked the distal extent of the trigger finger release. Sharp division of the A1 pulley was performed with a scalpel until the needle was encountered, thus completing the release. **Results:** A complete release of the pulley was achieved in all specimens with preservation of the A2 pulley. No digital nerve or artery injuries were noted with open inspection of the flexor sheath. **Conclusion:** Percutaneous marking of the distal extent of the A1 pulley is a safe and reliable method that not only ensures complete release of the A1 pulley but also preserves the A2 pulley. The placement of a small gauge needle adds no morbidity to this minimally invasive technique.

Tendon entrapment of the fingers and thumb is a common cause of hand pain and disability.^1^ Several forms of treatment of trigger finger have been reported. Nonsurgical modalities include steroid injection and splinting. Operative management focuses on surgical release of the entrapped tendon by either percutaneous or open approach.^2^–^6^ Multiple types of incisions have been described^7^–^9^; however, division of the A1 pulley is not without complications. Digital nerve injury, inadvertent release of the A2 pulley, and bowstringing are infrequent but significant causes of postoperative morbidity. Bowstringing after A2 pulley injury is manifested as a protrusion of the flexor tendon into the palm during finger flexion. It can cause a painful pulling sensation in the palm with associated failure to fully extend or flex the finger actively.^10^

Previous studies have attempted to clarify the complexity of the digital pulley system and its nomenclature.^11^–^15^ A recent anatomic study^16^ has allowed surgeons to predict the location of the A1 pulley on the basis of hand surface landmarks. On the basis of these measurements, we present a new technique to circumvent injury to the A2 pulley during a trigger finger release. It is a simple, practical, and inexpensive method, which assures complete incision through the A1 pulley while avoiding an injury to the A2 pulley on the basis of anatomic landmarks of the palm.

## SURGICAL APPROACH

**Figure 1:**
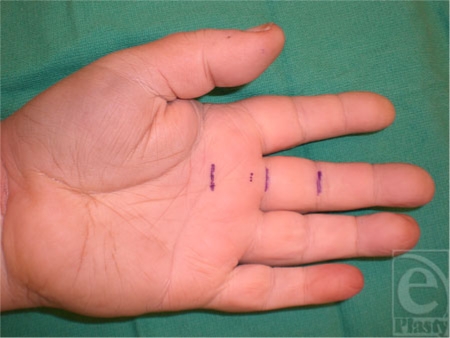
Hand surface landmark ratio of proximal interphalangeal distance to palmar digital crease (PDC) distance, used to predict the proximal A1 pulley edge. The distal edge of the A1 pulley is predicted 5 mm proximal to the PDC.

**Figure 2:**
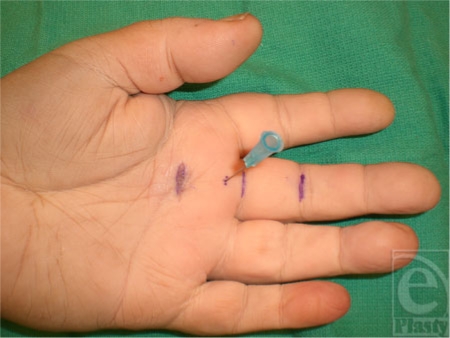
Placement of a 25-gauge needle at the distal edge of the A1 pulley.

**Figure 3:**
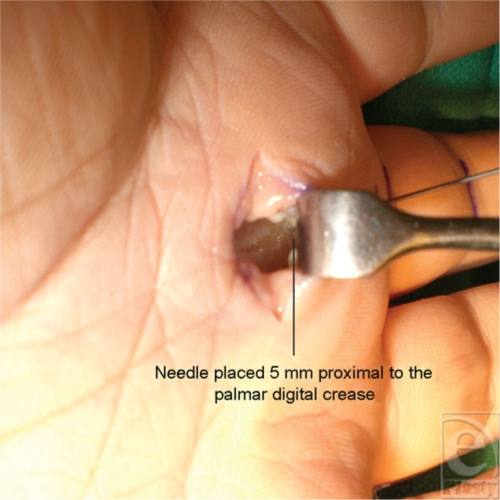
Sharp division of the A1 pulley extending to the percutaneous needle.

Nine fresh cadaveric hands were dissected with the aid of loupe magnification. The skin was marked based on known anatomic landmarks for trigger finger release as described earlier by Wilhelmi et al.^16^ A hand surface landmark ratio of the proximal interphalangeal (PIP) crease to palmar digital crease (PDC) distance was used to predict the proximal edge of the A1 pulley (Fig 1). On average, the PIP to PDC distance was 2.42 cm for the index, long, and ring fingers. After measurement of the PIP to PDC distance, an equal distance was marked proximal to the PDC. The proximal portion of the cruciate (C0) pulley defined the distal edge of the A1 pulley at 0.46 cm proximal to the PDC. Percutaneous placement of a 25-gauge needle 5 mm proximal to the PDC marked the distal extent of the release (Fig 2). A transverse incision was made overlying the proximal edge of the A1 pulley, allowing clear visualization of the anatomy. The pulley release was performed sharply with a scalpel until the 25-gauge needle was encountered, thus completing the release (Fig 3).

**Figure 4:**
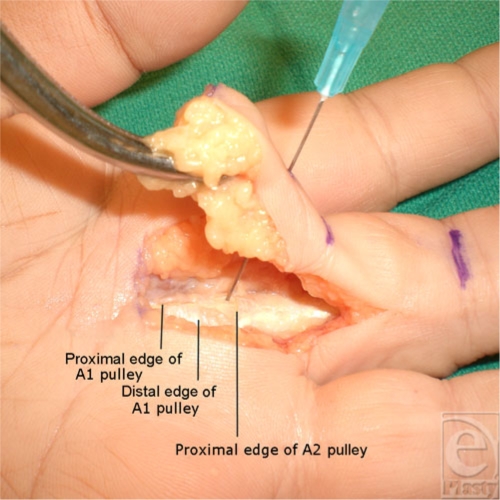
Exposure of the flexor sheath demonstrating complete division of the A1 pulley with preservation of the A2 pulley.

On inspection of the flexor tendon sheath, no arterial or nerve damage was noted in all the specimens. The proximal edge of the A2 pulley was fully preserved in all specimens as demonstrated in Figure 4.

Care must be taken when attempting percutaneous release of the border fingers.^17^–^19^ The oblique course of the flexor tendons and neurovascular structures of the index and small fingers pose a greater challenge. Longitudinal landmarks of the scaphoid tubercle to the midline of the small finger and the pisiform to the midline of the index finger can be used to mark the course of the flexor tendon to the border fingers.^20^ A modification of the marking pertains to the small finger and its relatively short A1 pulley, averaging 0.98 cm.^20^ Placement of the percutaneous needle should follow the PIP to PDC distance measurements while taking into account the shorter length of the pulley.

## SUMMARY

Knowledge of anatomical landmarks of the hand can assist with demarcating the distal extent of the A1 pulley release. Percutaneous placement of a 25-gauge needle 5 mm proximal to the PDC marks the distal edge of the pulley and prevents injury to the A2 pulley. It is a safe and reliable method, which adds no morbidity to this minimally invasive technique.
